# Mechanical and Thermal Properties of AlN-SiC Composite Ceramics Fabricated by In Situ Reaction Hot-Pressing Sintering

**DOI:** 10.3390/ma19112263

**Published:** 2026-05-27

**Authors:** Xiaoqing Zhao, Bin Wang, Ping He, Shuaihang Qiu, Xiaoshuo Zhang, Weizhou Xin, Jinbao Pang, Run Huang

**Affiliations:** 1 School of Materials Science and Engineering, Anhui University of Science and Technology, Huainan 232001, China; zhaoxq@aust.edu.cn (X.Z.);; 2 State Key Laboratory of Advanced Ceramics, Shanghai Institute of Ceramics, Chinese Academy of Sciences, Shanghai 200050, China; 3 Anhui Industrial Generic Technology Research Center for New Materials from Coal-Based Solid Wastes, Huainan 232001, China; 4 State Key Laboratory of Advanced Technology for Materials Synthesis and Processing, Wuhan University of Technology, Wuhan 430070, China; shuaihangqiu@whut.edu.cn

**Keywords:** AlN-SiC composites, in situ reaction hot pressing, mechanical properties, thermal conductivity

## Abstract

**Highlights:**

- Fine-grained and dense AlN-SiC ceramics were fabricated at relatively low temperatures.
- The Si additive melted and wetted the AlN matrix via capillary action, providing sufficient sintering driving force.
- Carbon removed oxygen impurities to purify grain boundaries and in situ reacted with liquid Si to form lamellar SiC.
- Uniform SiC particles at grain boundaries exerted strong pinning effects to maintain fine submicron AlN grains.
- The synergy of high density, refined grains and clean interfaces endows ceramics with excellent performance.

**Abstract:**

Simultaneously achieving high densification, excellent mechanical properties, and high thermal conductivity remains challenging for aluminum nitride–silicon carbide (AlN-SiC) composites. In this study, fine-grained AlN-SiC composite ceramics were fabricated via in situ reaction hot pressing with the addition of small amounts of silicon (Si) and carbon (C). At an optimal sintering temperature of 1800 °C, the primary phase composition consisted of AlN, SiC and residual graphite, with an average AlN grain size of 0.94 μm. The Si additive melted and wetted the AlN matrix via capillary action, thereby providing sufficient kinetic driving force for densification. Meanwhile, the C additive not only removed oxygen impurities and purified grain boundaries but also reacted in situ with liquid Si to form SiC. The uniformly dispersed SiC particles inhibited the abnormal growth of AlN grains via the grain boundary pinning effect. Consequently, the relative density, flexural strength, and Vickers hardness of the obtained AlN-SiC ceramics reached 99.08%, 365 MPa and 22.58 GPa, respectively. At room temperature, the composite exhibited a thermal conductivity of 66 W/(m·K) and a thermal diffusivity of 32.6 mm^2^/s. This superior thermal performance is attributed to the purified grain boundaries, uniform SiC distribution, high densification, and tightly bonded SiC/AlN interfaces, which result in weak phonon interfacial scattering.

## 1. Introduction

With the rapid advancement of electronic devices toward higher power densities and greater integration, significant heat generation during operation has become a critical concern. Inefficient heat dissipation severely compromises device stability and service life [1]. Therefore, the development of ceramic substrates possessing high thermal conductivity, excellent mechanical properties, and reliable environmental stability has become a research priority [2]. Aluminum nitride (AlN) ceramics are regarded as ideal heat-dissipation materials to replace toxic beryllium oxide (BeO), owing to their high theoretical thermal conductivity (~320 W/m·K), low dielectric constant (*ε* ≈ 8~10), and thermal expansion coefficient (~4.5 × 10^−6^ K^−1^) that closely matches that of silicon chips [3]. However, AlN is a typical strongly covalent compound characterized by a low atomic self-diffusion coefficient and a high melting point (>2200 °C), which renders densification difficult. Moreover, its mechanical properties require improvement to meet the structural stability demands of high-power devices. Furthermore, AlN particles are prone to oxidation, forming an oxide layer on their surface [4,5], which significantly hinders phonon conduction and results in an actual thermal conductivity far below the theoretical value.

Currently, the use of sintering additives such as yttrium oxide (Y_2_O_3_), yttrium fluoride (YF_3_), cerium oxide (CeO), and samarium oxide (Sm_2_O_3_) is considered an effective approach to address the sintering challenges of AlN ceramics [6,7,8]. For instance, Huang et al. [9] fabricated dense AlN ceramics via pressureless sintering at 1850 °C using multiple oxide additives. The resulting aluminate liquid phase promoted particle rearrangement and densification, reducing the sintering temperature. With Y_2_O_3_ as the additive, the sintered AlN ceramics exhibited the highest thermal conductivity (205 W/(m·K)) and flexural strength (295 MPa). Wei et al. [10] also fabricated Y_2_O_3_-doped AlN ceramics using green bodies with varying oxygen (O) and carbon (C) contents, achieving a thermal conductivity of 221.64 W/(m·K) and a flexural strength of 397.16 MPa. Although liquid-phase sintering additives can significantly reduce the sintering temperature of AlN ceramics, the subsequent abnormal grain growth and notable reductions in thermal and mechanical properties are inherent drawbacks that are difficult to eliminate.

In this context, introducing a second phase with excellent thermal and mechanical properties to prepare high-performance AlN composite ceramics has become a preferred strategy [11]. Among them, silicon carbide (SiC) possesses high thermal conductivity (~490 W/m·K), excellent high-temperature mechanical properties, oxidation resistance and chemical stability [12,13]. Compositing AlN with SiC integrates the advantages of both materials and is expected to resolve the densification challenges of AlN while imparting superior overall performance.

Various methods for preparing AlN-SiC composites have been developed for applications in electronic packaging, high-temperature structural materials, microwave components, thermoelectric conversion elements, and thermal management systems [14,15,16,17,18,19]. Sun et al. [15] adopted a silicon–aluminum (Si-Al) alloy as the infiltration medium and porous silicon nitride (Si_3_N_4_) as the pre-infiltration substrate, successfully fabricating dense AlN-SiC composite ceramics via low-temperature reactive melt infiltration. When the residual silicon mass fraction was 4%, the composite exhibited a flexural strength of 320.1 MPa and a thermal conductivity of 26.3 W·m^−1^·K^−1^. Li et al. [16] obtained high-density AlN-SiC composites with different SiC contents (0~50 wt%) at 1800 °C via plasma-activated sintering. As the SiC content increased, the thermal conductivity gradually decreased, with the optimal value of 24.88 W·m^−1^·K^−1^ achieved at a SiC content of 30 wt%. Lu et al. [17] fabricated AlN-SiC composite ceramics at 2000 °C under nitrogen (N_2_) atmosphere through pressureless sintering with Y_2_O_3_ as the sintering additive. The maximum thermal conductivity reached 45.53 W·m^−1^·K^−1^. Roy et al. [18] investigated the thermal conductivity of AlN-SiC ceramics with Y_2_O_3_ as a sintering aid across multiple compositions. They reported that as the SiC content increased from 20 to 50 wt%, the thermal conductivity varied between 49.0 and 61.7 W·m^−1^·K^−1^.

Based on the above analysis, there are still many challenges in preparing AlN-SiC composites using existing technology. Conventional pressureless sintering makes it difficult to obtain high-density composites, and the presence of pores severely hinders heat conduction, resulting in low thermal conductivity [20]. Hot-pressing sintering method often requires extremely high sintering temperatures (>1950 °C), which not only consume substantial energy but also impose stringent demands on equipment [21]. In addition, the direct addition of SiC particles tends to cause particle agglomeration and weak interfacial bonding, which further degrades the material performance. Therefore, developing a novel preparation method for AlN-SiC composites that operates at relatively low sintering temperatures, requires no complex sintering additives, and simultaneously achieves high density, high thermal conductivity, and excellent mechanical properties holds significant application value.

In recent years, reactive hot-pressing sintering has emerged as a promising ceramic preparation technique and has been successfully applied in the fabrication of various nitride, carbide and boride ceramics, such as titanium nitride (TiN) [22], SiC [23], and titanium diboride (TiB_2_) [24], etc. This technique couples in situ chemical reactions, hot-pressing densification, and pressure-assisted mass transfer, which offers significant advantages in reducing the sintering temperature, generating clean interfacial phases in situ, and inhibiting grain growth [25]. Instead of the traditional approach of directly adding SiC as a second phase, this study adopts a strategy of incorporating small amounts of Si and C, which react in situ during the hot-pressing sintering process to generate SiC reinforcing phases within the AlN-SiC composite ceramics. The densification behavior, microstructure, mechanical properties, and thermal conductivity of the resulting composites were systematically investigated. This study provides technical support for the application of AlN-based ceramics in high-power electronic packaging and other fields.

## 2. Materials and Methods

Commercially available AlN powder (d_50_ = 0.7 μm, purity ≥ 99.9%, Shandong Sinocera Functional Material Co., Ltd., Dongying, China) was selected as the matrix material, while Si (purity ≥ 99.9%, Beijing Huawei Ruike Chemical Technology Co., Ltd., Beijing, China) and graphite powder (purity ≥ 99%, Beijing Huawei Ruike Chemical Technology Co., Ltd., Beijing, China) were used as the raw materials for the reactive sintering process to prepare AlN-SiC composite ceramics. The AlN, Si, and C powders were weighed in a mass ratio of 97:2:1. In this mixture, the Si content was slightly below the stoichiometric ratio for SiC formation, which requires a Si:C mass ratio of approximately 2.33:1. The excess carbon was intended to facilitate deoxidation and removal of impurities during sintering. Anhydrous ethanol was used as the ball milling medium. The mixture was ball-milled for 12 h in a drum ball mill. After dispersion and mixing, the solvent was removed using a rotary evaporator and then moved to a drying oven for 24 h to obtain the mixed powder. Subsequently, a certain amount of the mixture was weighed and placed into a graphite mold. The mold and the mixture were separated by graphite paper. Sintering was performed in a hot press furnace (ZT-40-21Y, Shanghai Chenhua Technology Co., Ltd., Shanghai, China) under a vacuum atmosphere. The samples were separately heated to 1700 °C, 1800 °C and 1900 °C, with a maximum sintering pressure of 30 MPa. For comparison, pure AlN ceramics were also prepared under the same conditions.

**Figure 1:**
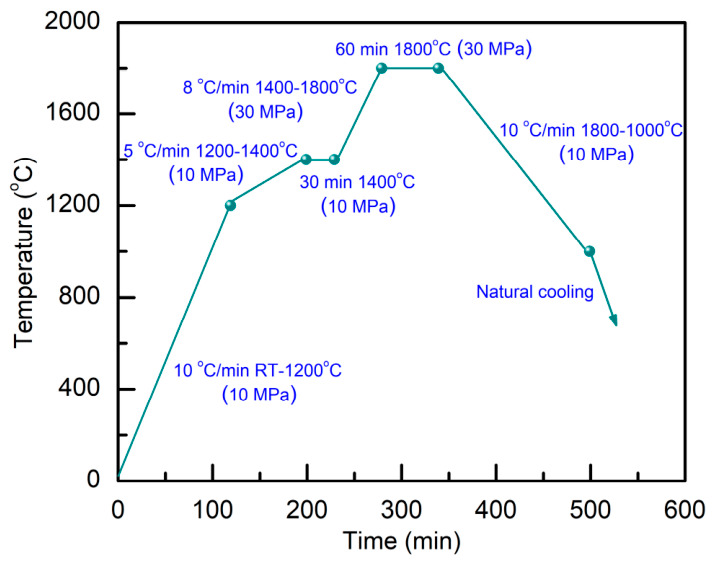
The sintering procedure for the AlN-SiC composite ceramics.

Since the sintering and pressure conditions critically influence the performance of this system, in this study, the sintering procedure for the AlN-SiC composite ceramics is presented in Figure 1. The initial pressure was set to 10 MPa, and the temperature was raised to 1200 °C at a heating rate of 10 °C/min. Then, the temperature was further increased to 1400 °C at a heating rate of 5 °C/min and held for 30 min, while the pressure was maintained at 10 MPa. Next, the pressure was increased to 30 MPa, and the temperature was raised to the target sintering temperature (1700–1900 °C) at a heating rate of 8 °C/min, followed by a holding period of 60 min. To prevent the generation of large residual stresses during the cooling process, a cooling procedure was set up. The pressure was reduced to 10 MPa, and the temperature was decreased to 1000 °C at a rate of 10 °C/min. Finally, the pressure was fully released, and the sample was allowed to cool naturally within the furnace to room temperature. In addition, the sintered samples were machined to the specified dimensions for subsequent tests using a three-axis water-jet guided laser cutting machine (FN-30X, Feina Laser Technology Co., Ltd., Ningbo, China).

The sample displacement during sintering was monitored using the data acquisition system of the hot-pressing furnace. After eliminating the thermal expansion effect of the graphite die, the sintering shrinkage curve was acquired. The time-dependent shrinkage rate was further calculated by differentiating the displacement curve. The bulk density of the sintered samples was measured using the Archimedes method with deionized water as the immersion medium. The relative density was calculated as the ratio of the measured bulk density to the theoretical density. Vickers Hardness (Hv) was measured on polished sample surfaces using a Vickers hardness tester (HV-30Z, Laizhou Huayin Testing Instrument Co., Ltd., Laizhou, China) with an applied load of 9.8 N and a dwell time of 15 s. The flexural strength was evaluated using a three-point bending test (MTS810, Eden Prairie, MN, USA). The phase composition of the sample was analyzed by X-ray diffraction (XRD-6000, Shimadzu Corporation, Tokyo, Japan) over a 2θ range from 20° to 80°. The microstructure and element distribution of the sample were examined using a field emission scanning electron microscope (FSEM, Zeiss Sigma 300, Oberkochen, Germany) equipped with an energy-dispersive X-ray spectroscopy (EDS) system. Thermodynamic calculations for the AlN-Si-C system were performed using FactSage 8.2 software to predict possible reactions and equilibrium phase compositions during high-temperature sintering. The calculations were conducted over a temperature range of 1200 °C to 1900 °C under a vacuum pressure of 0.1 Pa. The thermal conductivity and specific heat capacity were evaluated using a laser thermal analyzer (LFA457, Netzsch, Selb, Germany). To ensure accuracy, the specific heat capacity was also theoretically calculated using the Dulong-Petit law. According to this law, the molar heat capacity of a solid compound is given by *n*·3*R*, where *n* is the number of atoms per formula unit and *R* is the universal gas constant (8.314 J·mol^−1^·K^−1^). The mass-specific heat capacity (*C*_p_) is then obtained by dividing the molar heat capacity by the molar mass. The total thermal conductivity (κ_tot_) was calculated by Equation (1) [26].

(1) $$\kappa_{\mathrm{tot}}=C_{p}\times D\times \rho$$

where κ_tot_ is the total thermal conductivity (W·m^−1^·K^−1^), *D* is the thermal diffusivity, (m^2^·s^−1^), *C*_p_ is the specific heat capacity (J·kg^−1^·K^−1^), and *ρ* denotes the bulk density (kg·m^−3^).

## 3. Results and Discussion

**Figure 2:**
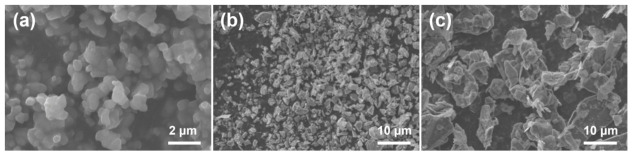
Morphologies of raw material powders: (**a**) AlN, (**b**) Si, and (**c**) graphite.

The initial morphology of the raw material powders significantly influences the densification behavior and resulting properties of the AlN-Si-C system during reactive sintering. Figure 2 shows the microstructures of the three raw material powders. The AlN powder exhibited a near-spherical shape and a uniform size distribution, with particle sizes ranging from 0.5 to 1.5 μm (Figure 2a). These morphological features contribute to its high surface energy. The Si powder consisted of irregular particles ranging in size from approximately 0.5 to 5 μm (Figure 2b). The graphite powder displayed a typical flaky layered structure (Figure 2c) and exhibited high reactivity, which enables its in situ reaction with Si powder to form SiC.

**Figure 3:**
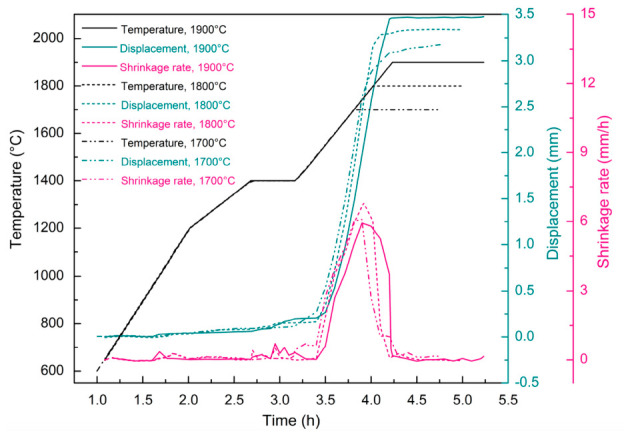
Sintering densification curves of samples at different temperatures.

The densification curves of the AlN-Si-C system obtained during hot-pressing sintering are presented at various temperatures in Figure 3. Below 1300 °C, the sample displacement showed no apparent change. At this stage, only initial particle contact occurred, while the reaction between Si and graphite had not yet initiated. At 1400 °C, the system temperature approached the melting point of Si, causing the Si powder to soften and form a liquid phase. The capillary forces of the liquid phase drove particle rearrangement, leading to initial densification, which is consistent with the liquid-phase sintering mechanism of AlN-based composite ceramics reported in previous studies [18]. Meanwhile, the in situ reaction between Si and graphite began, accompanied by slight shrinkage. From 1600 °C to 1900 °C, the displacement curve exhibited a steep rise, corresponding to the main densification stage of the sintering process. The shrinkage rate curve simultaneously rose sharply, reaching a peak in the range of 1700 °C to 1800 °C before rapidly decreasing. During this stage, Si and graphite in the AlN-Si-C system rapidly reacted in situ to form SiC [27], accompanied by significant reaction-induced shrinkage. Simultaneously, the AlN matrix underwent solid-state sintering at high temperatures, achieving densification through grain boundary and volume diffusion. During the isothermal hold at 1900 °C, the displacement curve reached a plateau, indicating that the densification process of the system was substantially complete. Comparison of the sintering curves at various temperatures reveals that the densification of the AlN-Si-C system was decisively affected by the sintering temperature and gradually increased with rising temperature.

**Figure 4:**
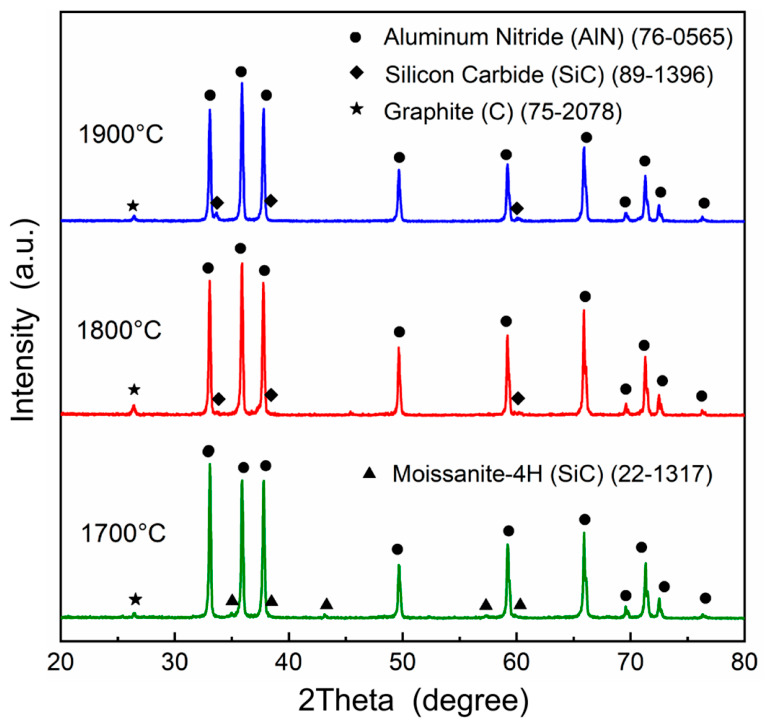
XRD patterns of AlN-SiC composites sintered at different temperatures.

The XRD patterns of AlN-SiC composites sintered at different temperatures are shown in Figure 4. With increasing sintering temperature, the primary crystalline phases in the AlN-SiC ceramics remained AlN, SiC, and a small amount of residual graphite, with no intermediate phases detected. This indicates a well-defined reaction pathway without byproduct formation, confirming the successful in situ generation of SiC reinforcement through the reaction between Si and graphite powders. This phase composition is consistent with previous reports on AlN-SiC composites [21]. At 1700 °C, the as-formed SiC predominantly exhibited a hexagonal crystal structure, with relatively weak characteristic peak intensities. Concurrently, a distinct graphite peak appeared at 2θ ≈ 26 °, which corresponds to unreacted carbon residues. According to the in situ reaction Si + C → SiC, the atomic masses of Si and C are 28 and 12, respectively. In the initial raw material proportions (AlN:Si:C = 97:2:1 by mass), the amount of carbon exceeded that required for complete reaction with silicon. Consequently, only part of the carbon participated in the SiC synthesis, and the remaining excess carbon was not fully consumed, finally existing as a residual graphite phase. This finding is consistent with the characteristic diffraction peaks of unreacted carbon observed in the XRD patterns. Upon increasing the sintering temperature to 1800 °C, new characteristic peaks associated with cubic SiC emerged, demonstrating that the elevated temperature not only promoted the reaction between Si and graphite but also induced a phase transformation of SiC. As the temperature was further increased to 1900 °C, no obvious change was observed in the main phase composition of the system, indicating that the phase composition of the composite stabilizes after reaching the optimal sintering temperature.

**Figure 5:**
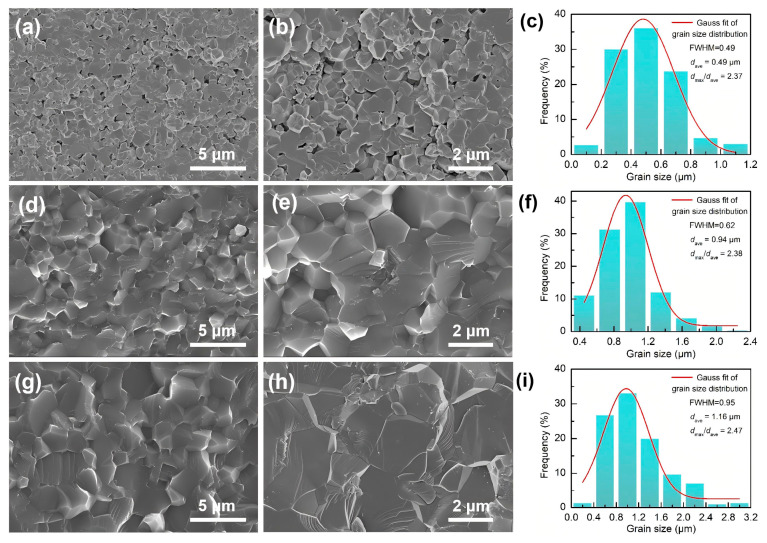
Morphologies and particle size distribution images of AlN-SiC ceramics sintered at different temperatures: (**a**–**c**) 1700 °C, (**d**–**f**) 1800 °C, and (**g**–**i**) 1900 °C.

The morphologies of fracture surfaces at different sintering temperatures for AlN-SiC composites are presented in Figure 5. Numerous closed pores were evident on the fracture surface of the AlN-SiC composite ceramic sintered at 1700 °C, indicating that full densification was not achieved, and only sintering necks had developed between grains (Figure 5a,b). The AlN grains displayed fine and equiaxed shapes with a relatively uniform size distribution. As shown in the grain size distribution in Figure 5c, the grain sizes predominantly fell within the range of 0.2~0.8 μm, exhibiting an approximately normal distribution with an average grain size of 0.49 μm. Neither an obvious bimodal distribution nor abnormally large grains were detected. The full width at half maximum (FWHM) serves as an indicator of the uniformity of the grain size distribution, with smaller values reflecting greater uniformity. The d_max_/d_ave_ ratio provides a quantitative measure for assessing abnormal grain growth, where a value below 2.5 suggests the absence of such growth. For the sample sintered at 1700 °C, the FWHM of the AlN grains was 0.49 μm, and the d_max_/d_ave_ ratio was 2.37, implying that no significant grain growth occurred at this temperature.

Figure 5d,e show SEM images of the AlN-SiC composite ceramic sintered at 1800 °C. The microstructure was dense and homogeneous, with no visible pores or cracks, indicating that near-full densification was achieved. The AlN grains exhibited well-developed polyhedral morphologies with tightly bonded grain boundaries, which is characteristic of high-performance AlN-based composite ceramics [28]. The fracture surface exhibited a mixed mode of transgranular and intergranular fracture. According to the grain size distribution in Figure 5f, the average grain size of the sample was 0.94 μm, with a uniform distribution and no obvious abnormal grain growth, representing a typical fine-grained dense microstructure. Driven by the elevated temperature, the Si additive melted and thoroughly wetted the AlN matrix via capillary action, providing sufficient kinetic driving force for densification. In addition, the C additive not only eliminated oxygen impurities and purified the grain boundaries within the AlN matrix, but also reacted in situ with liquid Si to generate SiC as a secondary phase [29]. The in situ formed SiC particles were uniformly dispersed along the AlN grain boundaries, which effectively inhibited abnormal grain growth at high temperatures through the grain boundary pinning effect [30]. Consequently, even at high sintering temperatures, the material preserved fine and uniform submicron grains. Additionally, the axial pressure applied during hot pressing significantly facilitated particle rearrangement, pore elimination, and grain boundary sliding, which suppressed defects such as pores and cracks caused by volume changes from the in situ reaction, thereby preventing structural loosening [31]. The densification temperature achieved in this in situ reaction sintering is significantly lower than that reported for AlN-SiC composite ceramics prepared by conventional methods (e.g., direct mixing of AlN and SiC powders) [18,20,32]. As shown in Figure 5g,h, the grains were in close contact with no visible porosity; however, pronounced grain growth occurred in the AlN-SiC ceramic at 1900 °C. The excessively high temperature dramatically accelerated the grain boundary migration rate [33]. Under such conditions, despite the applied hot pressing pressure still maintaining a relatively high density, the pinning effect exerted by the in situ formed SiC particles on the AlN grain boundaries was substantially weakened. As a result, the average grain size increased to 1.16 μm, the d_max_/d_ave_ ratio rose to 2.47, and noticeable grain growth was accompanied by a broadening of the grain size distribution (Figure 5i).

**Figure 6:**
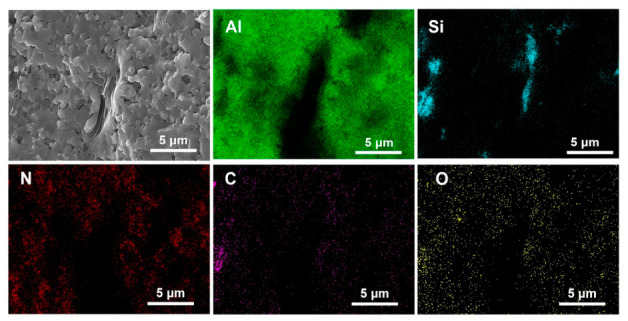
Morphology and elemental mapping images of AlN-SiC ceramics sintered at 1700 °C.

**Figure 7:**
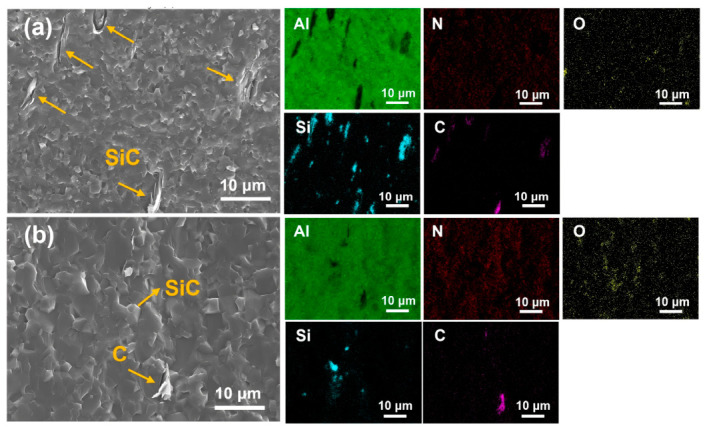
Morphology and elemental mapping images of AlN-SiC ceramics sintered at different temperatures: (**a**) 1800 °C and (**b**) 1900 °C.

To further elucidate the microstructural evolution of the AlN-SiC composite with sintering temperature, the morphology and elemental distribution maps of the sample sintered at 1700 °C are presented in Figure 6 and Figure 7. Al and N elements were uniformly co-located throughout the matrix, corresponding to the formation of the primary AlN phase. By contrast, Si and C elements showed pronounced local enrichment, indicating that the in situ reaction between Si and C had already commenced at 1700 °C (Figure 6), resulting in the formation of a lamellar SiC secondary phase. Notably, some graphitic carbon remained unreacted within the AlN-SiC ceramic. The resulting SiC lamellae featured an aspect ratio of approximately 3.60, closely resembling the morphology of the flaky graphite in the starting raw materials. This observation points to a template effect governing the formation process, which has also been confirmed in our previous study [34]. The templating effect of carbon can lead to the formation of high-aspect-ratio SiC structures. Specifically, Si melted at elevated temperatures to form a liquid phase during hot-pressing sintering, which was then forced under applied pressure to infiltrate and wet the surfaces and grain boundaries of AlN particles. Because this reaction takes place on the graphite surface, the lamellar morphology of graphite was preserved, yielding high-aspect-ratio, flake-like SiC. In addition, the oxygen signal was diffusely distributed across the fracture surface, indicating that the oxygen impurities in the raw materials were not sufficiently reduced by the carbon source at 1700 °C. Such impurities tend to form low-melting-point glassy phases at the grain boundaries, which not only compromise grain boundary bonding strength but also impair the high-temperature performance of the material [35].

Upon increasing the sintering temperature to 1800 °C, the fracture surface of the sample developed a dense microstructure. The enrichment of Si and C elements revealed that the in situ-formed lamellar SiC secondary phase was uniformly dispersed throughout the AlN matrix, effectively inhibiting abnormal grain growth of AlN (marked by yellow arrows in Figure 7a). Notably, carbon was locally confined to the SiC phase region, indicating that the majority of the carbon had been consumed by in situ reactions. On the one hand, it reacted with molten Si to generate the SiC secondary phase; on the other hand, it underwent a reduction reaction with oxygen impurities in the raw materials, generating CO/CO_2_ gases [36]. As a result, oxygen impurities were effectively removed from grain boundaries, the formation of low-melting-point glassy phases was curtailed, and grain boundary bonding strength was markedly enhanced. Consistent with this, the oxygen signal exhibited a diffuse distribution with no discernible grain boundary segregation, further confirming that oxygen impurities had been sufficiently reduced, yielding high-purity grain boundaries. These microstructural features provide the foundation for the material’s superior performance.

When the temperature was further raised to 1900 °C, however, a marked decrease in the number of lamellar SiC particles was observed, with only a small number of particulate SiC and lamellar carbon structures on the fracture surface (marked by yellow arrows in Figure 7b).The excessively high temperature induced a morphological transformation of SiC, which weakened its pinning effect on AlN grain boundaries and rendered it incapable of effectively restraining AlN grain growth (Figure 7b). Meanwhile, EDS analysis detected residual graphitic carbon, primarily attributable to the volatilization loss of Si at elevated temperatures. This loss disrupted the stoichiometric balance of the Si–C reaction, leaving a portion of the carbon unreacted. A comparison of the microstructural evolution across different temperatures identifies 1800 °C as the optimal sintering temperature for balancing in situ reaction and densification in the AlN–Si–C system. At this temperature, a lamellar SiC secondary phase was formed in situ, providing dispersion strengthening to the AlN matrix while simultaneously yielding a dense microstructure. It has also been reported that secondary phases with tailored microstructures can enhance the mechanical properties of the matrix [37]. Moreover, the residual carbon effectively purifies grain boundaries, a critical factor in ensuring outstanding mechanical and thermal properties.

**Figure 8:**
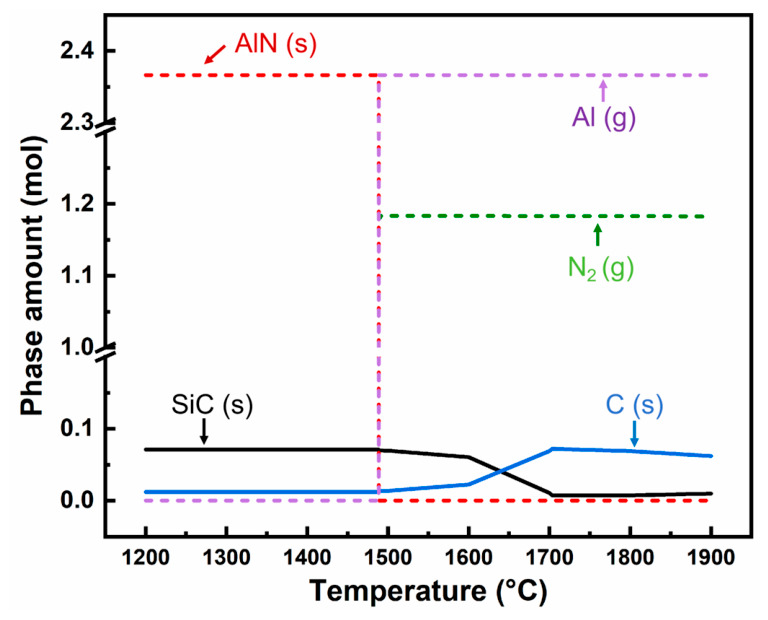
Thermodynamic equilibrium phase composition of the AlN-Si-C system as a function of temperature.

Thermodynamic equilibrium calculations for the AlN–Si–C system under a vacuum of 0.1 Pa over the temperature range of 1200 °C to 1900 °C are presented in Figure 8. According to these calculations, SiC can form spontaneously under thermodynamic equilibrium conditions and remain stable at temperatures exceeding 1200 °C (Figure 8), indicating that the in situ reaction between Si and C possesses a thermodynamic driving force even at relatively low temperatures. This finding also rationalizes the observation of SiC phases in samples sintered at various temperatures in this study. Furthermore, the thermodynamic calculations predicted the persistent presence of a small amount of residual carbon, which is consistent with the XRD (Figure 4) and SEM (Figure 6 and Figure 7) results obtained from the system.

The calculations further indicate that AlN began to decompose extensively into Al (g) and N_2_ (g) at temperatures above 1500 °C, while the equilibrium content of SiC gradually diminished above 1600 °C and dropped to very low levels beyond 1700 °C (Figure 8). Nevertheless, in the actual hot-pressing sintering experiments conducted in this study, no obvious decomposition of either the AlN matrix or the formed SiC phase was observed, apart from a slight reduction in the content of flake-like SiC grains at 1900 °C. This can be primarily attributed to the limited holding time of only 30 min at the maximum sintering temperature, which effectively restricted high-temperature decomposition [38]. In addition, the formation of a dense AlN solid skeleton contributed to the stabilization of both AlN and SiC by trapping any gaseous decomposition products within grain boundaries or closed pores.

**Figure 9:**
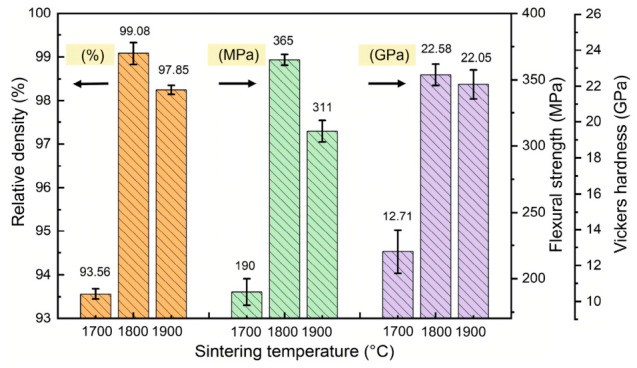
Relative density, flexural strength and Vickers hardness of AlN-SiC ceramics sintered at various temperatures.

Sintering temperature is a key determinant of both densification behavior and mechanical properties [39]. Figure 9 summarizes the relative density, flexural strength, and Vickers hardness of AlN-SiC composite ceramics sintered at different temperatures. All three performance indicators followed a consistent trend and peaked at 1800 °C. At 1700 °C, the relative density was merely 93.56%, and the sample contained numerous closed pores, indicating insufficient sintering driving force and limited atomic diffusion. Raising the temperature to 1800 °C dramatically increased the relative density to 99.08%, corresponding to near-full densification. This marked improvement is attributed to enhanced particle rearrangement, grain boundary sliding, and in situ reaction sintering, which effectively reduced the densification temperature [40]. In contrast, a further increase to 1900 °C led to a slight reduction in density, down to 97.85%. This decline is primarily caused by excessively rapid grain boundary migration, which traps residual pores as intragranular closed pores during abnormal grain growth. Additionally, minor decomposition of AlN at elevated temperatures may introduce gas defects, both factors contributing to the reduced density. For pure AlN ceramics sintered at 1800 °C, the relative density was only 92.80%, confirming that the in situ formation of SiC grains within the AlN matrix via reaction sintering significantly lowered the densification temperature.

Consistent with the densification behavior, both flexural strength and Vickers hardness of AlN-SiC ceramics reached their maximum values at 1800 °C (Figure 9), i.e., 365 MPa and 22.58 GPa, respectively. These values are significantly higher than those of pure AlN ceramics sintered at the same temperature, which exhibited a flexural strength of 263 MPa and a Vickers hardness of 14.60 GPa. At 1700 °C, the AlN-SiC ceramics exhibited low density and high porosity. The abundant closed pores acted as stress concentration sites, promoting crack propagation under external loading, while the incompletely densified grain boundaries resulted in weak bonding. Consequently, the flexural strength and Vickers hardness were only 190 MPa and 12.71 GPa, respectively. At 1800 °C, the near-fully dense microstructure eliminated most pore-related defects. Grain boundaries became tightly bonded owing to the combined effects of applied hot-pressing pressure and in situ reactions. Meanwhile, the in situ-formed lamellar SiC phase was uniformly dispersed along the grain boundaries, exerting both grain boundary pinning and dispersion strengthening effects that effectively impede crack propagation. At 1900 °C, despite the relative density remaining high, significant AlN grain growth occurred, which weakened the fine-grain strengthening effect. Moreover, the number of lamellar SiC particles decreased, reducing the pinning effect and grain boundary bonding strength. Coupled with the presence of intragranular closed pores, these factors caused the flexural strength to drop to 311 MPa and the Vickers hardness to decline to 22.05 GPa (Figure 9). Thus, the optimal sintering temperature was 1800 °C, which achieved a balance among densification, microstructural uniformity, and mechanical properties.

**Figure 10:**
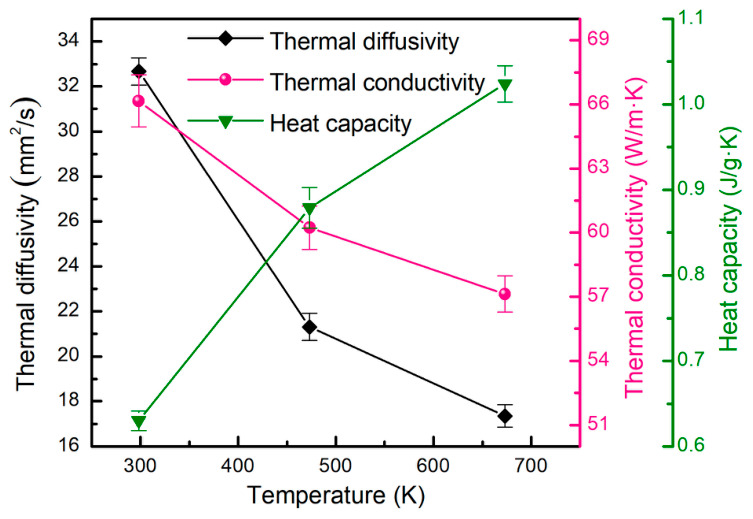
Temperature-dependent thermal property curves of AlN-SiC ceramics sintered at 1800 °C.

The in situ-formed SiC phase inevitably influences the thermal properties of the AlN matrix composite. Figure 10 displays the temperature dependence of thermal diffusivity, specific heat capacity, and thermal conductivity for the AlN-SiC composite sintered at 1800 °C over the range of 300–673 K. With increasing testing temperature, both thermal diffusivity and thermal conductivity decreased markedly, whereas specific heat capacity gradually increased. This behavior stems from enhanced lattice vibrations at elevated temperatures, which intensify phonon scattering and shorten the phonon mean free path [41]. At room temperature, the composite exhibited excellent thermal performance, achieving a thermal conductivity of 66 W/(m·K) and a thermal diffusivity of 32.60 mm^2^/s. At 673 K, the thermal diffusivity fell to 17.30 mm^2^/s, and the thermal conductivity dropped to 57 W/(m·K).

The thermal conductivity of the AlN-SiC composite prepared in this study was slightly lower than that of pure AlN ceramics (67.90 W/(m·K), due to additional phonon scattering caused by lattice mismatch and interfaces between the SiC phase and AlN matrix [42,43]. Nevertheless, the thermal conductivity achieved in this work remained considerably higher than the values of AlN-SiC ceramics reported in previous studies, such as those by Sun et al. (26.30 W/(m·K)) [15], Li et al. (24.88 W/(m·K)) [16] and Lu et al. (45.53 W/(m·K)) [17]. The processing conditions and properties of the AlN-SiC composite are summarized in Table 1. This superior thermal performance can be attributed to several factors. First, the uniformly distributed in situ-formed SiC phase avoids defect agglomeration. Second, the high densification degree, together with clean grain boundaries, reduces barriers to heat conduction. Third, the tightly bonded interfaces between SiC and AlN give rise to relatively weak phonon interfacial scattering. As a result, the composite retains high thermal conductivity despite the introduction of the SiC reinforcement phase.

## 4. Conclusions

In summary, AlN-SiC composite ceramics were successfully fabricated via in situ reactive hot pressing with the addition of Si and C. At a sintering temperature of 1800 °C, the composite achieved a relative density of 99.08%, a flexural strength of 365 MPa, and a Vickers hardness of 22.58 GPa. The uniformly dispersed lamellar SiC particles effectively inhibited abnormal AlN grain growth, thereby enhancing the mechanical properties. The composite also exhibited high thermal conductivity, with a room-temperature thermal conductivity of 66 W/(m·K) and a thermal diffusivity of 32.6 mm^2^/s, although both decreased at elevated temperatures due to intensified phonon scattering. This superior overall performance stems from the synergistic combination of high densification, a fine-grained microstructure, uniform SiC dispersion, clean grain boundaries, and weakly scattering SiC/AlN interfaces. This study provides a promising pathway for the design and fabrication of high-performance AlN-based composites for advanced thermal management applications.

## Figures and Tables

**Table 1:** Summary of the processing and properties for AlN ceramics.

AlN Ceramic	Processing	Vickers Hardness (GPa)	Flexural Strength (MPa)	Thermal Conductivity (W/(m·K))	Reference
AlN	HP 1800 °C	14.60	263	67.90	Present work
AlN-SiC	HP 1800 °C	22.58	365	66	Present work
AlN-SiC	Low temperature melt infiltration −1400 °C	16.90	320.10	26.30	[15]
AlN-SiC	SPS 1800 °C	/	/	24.88	[16]
AlN-SiC	PS 2000 °C	/	/	45.53	[17]

## Data Availability

The original contributions presented in this study are included in the article. Further inquiries can be directed to the corresponding authors.
